# Identifying Factors to Improve Oral Cancer Screening Uptake: A Qualitative Study

**DOI:** 10.1371/journal.pone.0047410

**Published:** 2012-10-24

**Authors:** Fatemeh Vida Zohoori, Kamini Shah, Julie Mason, Janet Shucksmith

**Affiliations:** 1 Health and Social Care Institute, Teesside University, Middlesbrough, United Kingdom; 2 Tees Public Health, Stockton on Tees, United Kingdom; The University of Hong Kong, Hong Kong

## Abstract

**Aims:**

To engage with high risk groups to identify knowledge and awareness of oral cancer signs and symptoms and the factors likely to contribute to improved screening uptake.

**Methods:**

Focus group discussions were undertaken with 18 males; 40+ years of age; smokers and/or drinkers (15+ cigarettes per day and/or 15+ units of alcohol per week), irregular dental attenders living in economically deprived areas of Teesside.

**Results:**

There was a striking reported lack of knowledge and awareness of oral cancer and its signs and symptoms among the participants. When oral/mouth cancer leaflets produced by Cancer Research UK were presented to the participants, they claimed that they would seek help on noticing such a condition. There was a preference to seek help from their general practitioner rather than their dentist due to perceptions that a dentist is ‘inaccessible’ on a physical and psychological level, costly, a ‘tooth specialist’ not a ‘mouth specialist’, and also not able to prescribe medication and make referrals to specialists. Interestingly, none of the 18 participants who were offered a free oral cancer examination at a dental practice took up this offer.

**Conclusions:**

The uptake of oral cancer screening may be improved by increasing knowledge of the existence and signs and symptoms of oral cancer. Other factors that may increase uptake are increased awareness of the role of dentists in diagnosing oral cancer, promotion of oral cancer screening by health professionals during routine health checks, and the use of a “health” screening setting as opposed to a “dental” setting for such checks.

## Introduction

According to a report from Cancer Research UK [1], 5,410 new cases of oral cancer were diagnosed in 2007 in the UK, which indicated a rise in incidence of 60% from 1975. The report also showed no improvement in survival rates over those years [1]. Oral cancer has a low 5-year survival rate with rates of 50% or less [2]. Both alcohol consumption and tobacco use are known risk factors for oral cancer. The incidence of oral cancer is also associated with social and economic status [3]. Differences in the 5-year survival rates for oral cancer between the most affluent and the most deprived groups have been reported: the survival rate for the former is almost 14% higher than that for the latter [1].

Early detection and treatment of oral cancer, when lesions are small and localised, is believed to be the most effective means to improve survival and reduce hospital costs and duration of treatment [4]. If the disease is diagnosed in its initial stages, not only could the 5-year survival rate increase to up to 80% [5] but also the patient’s quality of life would improve as a result of less aggressive and mutilating treatment [6].

Several reports have shown that half of patients present with an advanced lesion when it is too late for successful treatment. A recent study at a London teaching hospital revealed that 37% of participants delayed seeking medical advice by more than 3 months following the self-discovery of symptoms of oral cancer. The study also found that 53% of participants waited a month before seeking help [7]. A study in Greece reported a delay time of up to 780 days from initial symptoms to definitive diagnosis; and 52% of oral cancer patients had a delay of more than 21 days [8]. The Department of Health has identified improving the behaviour of cancer patients in seeking help as a high priority. In order to develop an effective intervention to minimise patient delay, the factors influencing delay in seeking help should first be understood. A recent systematic review [9] concluded that the reasons for patient delay in reporting oral cancer were poorly understood and under-researched, although symptomatology (change in symptoms, persistence and pain) has been suggested as one of the main triggers in a recent survey on barriers and triggers for seeking help by oral cancer patients [7]. Another cited contributing factor to possible delays in seeking treatment is lack of knowledge of the early signs of oral cancer. An earlier study of an at-risk population in the north-east of England (Newcastle upon Tyne), male drinkers and smokers over 44 years old, showed not only a lack of knowledge of the risks of oral cancer but also that they profess ignorance of the signs of oral cancer [10].

Although it is important to improve the awareness of all sectors of society, it is more crucial to target those where the incidence of disease is high. In order to develop effective approaches to meet this goal, the attitudes and beliefs of individuals at high risk should be understood first and then this knowledge used to design an effective intervention that will facilitate early diagnosis. Therefore, the aims of this study were to i) engage with high risk groups to identify knowledge and awareness of oral cancer signs and symptoms and ii) identify the factors which might contribute to improved screening uptake.

## Methods

### Ethics Statement

This study was conducted according to the guidelines laid down in the Declaration of Helsinki and all procedures involving human subjects/patients were approved by the School of Health and Social Care research ethics and governance committee, Teesside University (Ethics number: 058/10). Written informed consent was obtained from all participants.

A qualitative focus group methodology, as a well-established approach in medical and dental research, was employed for this study to identify, explore and explain complex attitudes and perceptions [10].

The study participants were recruited on the street in Middlesbrough by a team of trained and experienced market research interviewers, in accordance with the code of conduct of the Market Research Society [11], [12] using a pre-designed recruitment questionnaire, which included questions relevant to age, socio-economic status, frequency of smoking, drinking and visiting a dentist (Table 1). The inclusion criteria were: males over 40 years of age, irregular attenders for dental check-up, resident in economically deprived areas of Teesside, alcohol consumption exceeding 15 units a week and/or smoking over 15 cigarettes per day.

**Table 1:** Recruitment questionnaire: inclusion and exclusion criteria.

Question	Inclusion	Exclusion
Approaching participants: Gender	Male	Female
Q1. Attendance at a focus group discussion. If yes, when	No/Over 6 months ago	Yes/In the past 6 months
Q2. Age (year)/date of birth	40+	<40
Q3. Area of residency/post code (to be checked with thelist of eligible postcodes)	Residing in economically deprivedareas of Teesside	Residing in non-deprived areas
Q4 and Q5. Smoking and drinking: how often andhow many	Smoking 15+ cigarettes per day and/or drinking15+ units of alcohol per week	Smoking of <15 cigarettes per day and/ordrinking <15 units of alcohol per week
Q6. Visiting a dentist: frequency and reason	Visiting irregularly/Not a cancer related problem	Visiting a dentist: concerning mouth cancer

Eligible volunteers were informed of the general subject, and were then given an appointment card and information leaflet if they were interested in taking part in the study. They were then divided randomly into two groups. Discussion areas/topics (Table 2) were developed in advance as a guideline for use at the focus groups and directed by a highly experienced group mentor, allowing respondents to discuss issues free from observer bias or interference and encouraging interaction between group members. The focus groups lasted for almost 90 minutes. With the permission of the participants, the discussions were tape recorded and were then transcribed. At the end of focus group sessions, each participant was given a free mouth check-up voucher to use within 6 months of issue at the Teesside University dental practice.

**Table 2:** Brief summary of focus group discussion guide.

Introductions
Objective: To welcome participants, outline the purpose of the focus group discussion and how data would be used, and ask participants to introduce themselves.
**Lifestyle/Health**
Objective: To prepare the participants gently and set the tone before moving to the main topic of the discussion
**Oral Cancer – Spontaneous Association & Knowledge**
Objective: To find out the extent of spontaneous knowledge of participants about oral cancer and its signs/symptoms.
**Oral Cancer – Prompted Association & Knowledge**
Objective: To present printed materials (most common available leaflets/posters on oral cancer) to assess participants response to the content, words and imagery.
**Seeking Help**
Objective: To determine existing relationship/views of the participants with/on health care professionals.
**Encouraging Factors for Screening:** Belief about screening, Motivation to attend for screening and Fear of screening
Objective: To find out all barriers to gain access to services.

## Results

The characteristics of the group are presented in Table 3. There was a mix of workers and non-workers within the focus groups. Some participants regarded their general health as average, while some viewed their health more negatively.

**Table 3:** Characteristics of participants (n = 18) in Focus Groups.

Gender	Male	18
	Female	0
Age	Mean	48 year
	Range	40–61 year
Employment:	Unemployed	10 participants
	Employed (manual)	8 participants
Last visit to a dental surgery:	Within the last two years	6 participants
	Over two years ago	12 participants

*(S18- 45y, employed, irregular dental attenders) “Average…because I don’t exercise or anything like that so I’m not fully fit.”*

*(S03- 52y, unemployed, irregular dental attenders) “Knackered…all the abuse I have gave my body over the years.”*

Most participants perceived drinking as a social activity, and saw no harm in this. For some, drinking was deeply rooted in their life, with them evincing the argument that one would have ‘no life’ if their lifestyle was restricted to the extent that government health advice seems to suggest. They rejected a culture of being told ‘what not to do’.

*(S13- 50y, employed, irregular dental attenders) “I don’t worry about it. I’m not going to change me life around to avoid it.”*

*(S11- 48y, employed, regular dental attenders) “You have to go out and socialise sometimes or you will get stuck indoors.”*

The awareness of oral cancer was extremely low among the study participants:

*(S19- 41y, employed, regular dental attenders) “I’ve never known anyone with mouth cancer!”*

*(S12- 49y, employed, regular dental attenders) “Does anyone know about it in this room?”*

Only one respondent had some direct knowledge or experience of oral/mouth cancer (via a friend):

*(S19- 41y, employed, regular dental attenders) “Well funny enough, I was talking to a friend last week and he said ‘have you heard about a friend we know’, I said ‘no’. He said ‘he went to the dentist and he has got oral cancer’. He found out by going to the dentist for a check-up. Now I was like ‘what’s that then?’. I didn’t know what it was. He went in for a general check-up and came out with that!”*

There was some evidence of a passive or fatalistic attitude about developing cancer among the participants, and they believed that genetics and the environment play important roles in a person’s chance of developing oral cancer; only a few acknowledged that lifestyle could contribute in part:

*(S17- 47y, employed, regular dental attenders) “Anything can kick cancer off…the environment…air pollution…exhausts…heavy industry.”*

*(S16- 61y, unemployed, irregular dental attenders) “It’s in your genes.”*

*(S15- 57y, unemployed, irregular dental attenders) “Most people are at risk, 99.9%.”*

*(S18- 45y, employed, irregular dental attenders) “I don’t worry about it. I’ll go when my number’s up.”*

When they were asked to comment on things linked with oral/mouth cancer, their ‘guesses’ were: smoking, drinking, saliva, taking drugs, bad breath, rotten teeth and ulcers.

*(S08- 55y, unemployed, irregular dental attenders) “When you hear people talk about cancer they mention a lump or a growth, so you just assume if there is a lump or a growth (it’s cancer).”*

*(S15- 57y, unemployed, irregular dental attenders) “Teeth will fall out.”*

*(S02- 40y, unemployed, irregular dental attenders) “You’ll probably spit up blood.”*

*(S15- 57y, unemployed, irregular dental attenders) “Bad breath, obviously.”*

For the majority of the participants in the discussion groups, oral cancer was such a ‘new concept’, prior to talking about it as a group, that they would not have associated any of the above conditions with it.

*(S04- 43y, unemployed, irregular dental attenders) “I would never suggest if I had any of them I would think I had mouth cancer. Never in this world, you know what I mean? …. I would never think ‘oh I have mouth cancer’.”*

*(S14- 45y, employed, irregular dental attenders) “That’s the worse case scenario. We are not going to over react, that’s how men are, we just say it’s a little niggling pain.”*

The study participants had low to non-existent awareness of signs and symptom of oral cancer:

*(S04- 43y, unemployed, irregular dental attenders) “No, I wouldn’t (know what symptoms to look out for).”*

*(S09- 40y, unemployed, irregular dental attenders) “No, wouldn’t have a clue.”*

*(S14- 45y, employed, irregular dental attenders) “People are ignorant about it.”*

*(S20- 49y, employed, irregular dental attenders) “They don’t know because they haven’t got the information.”*

The groups were shown the available leaflets on oral cancer which illustrate the signs and symptoms of oral cancer. None of the participants had seen the leaflets before. Although the graphic pictures in the leaflets grabbed the attention of the participants, some regarded them as unnecessarily shocking:

*(S08- 55y, unemployed, irregular dental attenders) “It’s just disgusting…Not very nice pictures to look at.”*

*(S06- 41y, unemployed, irregular dental attenders) “I have never met anyone like that!”*

*(S04- 43y, unemployed, irregular dental attenders) “With a lip like that surely you have to go to the doctors and say ‘what’s the matter with my lip?’.”*

*(S13- 50y, employed, regular dental attenders) “Photographs definitely get through to you more.”*

Participants felt they were not qualified to check their own mouths for the early signs and symptoms of oral cancer. Indeed, they stated they would seek help from their general practitioner (GP) due to ‘ease of access’ as they were registered with the GP and, crucially, because they did not believe that a dentist had the right skills to check for signs of oral cancer. This was because they perceived a dentist as a ‘tooth specialist’, rather than a ‘mouth specialist’. They also believed (incorrectly) that a dentist lacked the power of a doctor to make referrals and write prescriptions:

*(S14- 56y, employed, irregular dental attenders) “I’m petrified of the dentist’s.”*

*(S09- 40y, unemployed, irregular dental attenders) “It’s a business, dentists, it’s a scam! I can’t see why you have to pay when you’re NHS.”*

*(S16- 61y, unemployed, irregular dental attenders) “You pay for a check-up at the dentist. You wouldn’t pay for a check-up at the doctor’s.”*

*(S15- 57y, unemployed, irregular dental attenders) “If I was having tooth problems I’d go to see the dentist”; “According to these pictures it’s nothing to do with your teeth, it’s more your gums, and you wouldn’t think because of your mouth or jaw go to the dentists.”*

*(S08- 55y, unemployed, irregular dental attenders) “Would a dentist be qualified? Because I just thought they dealt with teeth. Are they qualified?”*

*(S10- 44y, unemployed, regular dental attenders) “When you think cancer, you wouldn’t think of going to the dentist!”*

*(S12- 49y, employed, regular dental attenders) “I have regular visits to the dentist, go once every six months, and I’ve never had a problem in a long time. In regards to a doctor, I would rather see my doctor if I thought psychologically I had a problem that needed attention.”*

*(S20- 49y, employed, irregular dental attenders) “Because the doctor has the wider range of general knowledge, the dentist to me is more like a business and you don’t really know if he has the diagnosis skills to really assess what’s there. Theoretically he should be able to say ‘oh yes, that’s mouth cancer’, but it might be something totally different, whereas at least the doctor can give you a referral to a specialist if there is something seriously wrong.”*

*(S 16- 61y, unemployed, irregular dental attenders) “It would probably be better at a doctor’s than a dentist”; “They (GP’s) would be more experienced.”*

The participants were unaware of any oral cancer screening programme:

*(S06- 41y, unemployed, irregular dental attenders) “Nobody knows about it!”*

*(S01- 42y, unemployed, irregular dental attenders) “I know about breast cancer screening but not about the mouth.”*

The participants with a job would have welcomed being screened for oral cancer at work, whereas the non-workers would have preferred to be screened by their GP when they visit for other matters.

*(S15- 57y, unemployed, irregular dental attenders) “If you were to call at the doctors and they looked at the computer and realised you haven’t had your oral cancer check, they could take a swab.”*

*(S03- 52y, unemployed, irregular dental attenders) “Even the nurse or receptionist could say ‘by the way, you haven’t had this check in a while.”*

It was perceived that the screening should be cost free, quick, pain free and primarily visual which could be conducted by a trained medic:

*(S17- 47y, employed, regular dental attenders) “If it’s just a visual and a (possible) referral do you really need a doctor or a dentist to check that? Could you not get any trained medic to do that? If it’s just visual they can say ‘oh yeah, you need to be referred then pass you on to someone a bit more knowledgeable.”*

*(S04- 43y, unemployed, irregular dental attenders) “If it’s free and its quick people will do it.”*

It was stressed by the participants that a non-judgemental, ‘across-the-board’ attitude should be promoted, and that the message should be positive about maintaining a healthy mouth, rather than negative about cancer detection:

*(S03- 52y, unemployed, irregular dental attenders) “Don’t criticise my lifestyle. Just say ‘do it’ if you want to get tested.”*

*(S15- 57y, unemployed, irregular dental attenders) “You don’t want to be patronised for being a drinker or smoker, and for them to say ‘because this is your lifestyle you are more at risk than anybody else’.”*

Most participants argued that the most appropriate way to inform them about oral cancer and screening was through ‘personal interaction’ with their GP; and some admitted they rarely read literature in the waiting room of their GP surgery:

*(S06- 41y, unemployed, irregular dental attenders) “I have a rare visit to the doctor but I don’t really look at the leaflets.”*

*(S19- 41y, employed, regular dental attenders) “Next time you go to the doctor it should be kept on the records that you need a check.”*

Most participants claimed that they would attend screening if it was quick and easily available, but many objected to being screened in a dental surgery:

*(S18- 45y, employed, irregular dental attenders) “I wouldn’t do it (at the dentist).”*

*(S06- 41y, unemployed, irregular dental attenders) “If you go to a dentist you have to find a dentist you trust and, if he wants to accept you, firstly you want to find out why he has accepted you - not for the money, not because it’s free, who’s going to pay for it? But if you go to North Tees (local hospital) you have the doctors, nurses and qualified staff to look inside your mouth. I definitely think it’s better to walk into North Tees, to that wing of the hospital.”*

*(S20- 49y, employed, irregular dental attenders) “At the doctor’s wouldn’t bother me.”*

No participants subsequently used the provided free mouth check-up voucher to attend the check-up at the Teesside University dental practice.

## Discussion

This paper provides the first report on oral cancer awareness of ‘at risk’ males in Teesside. Although the present study was based on a relatively small sample size, the qualitative approach using focus group discussion to engage with the target population provided a thorough insight into the knowledge and awareness of oral cancer signs and symptoms among this group and enabled identification of the factors which might contribute to improved screening uptake. Based on the findings of this study, awareness of oral cancer among ‘at risk’ males in Teesside was low, in contrast with a relatively recent study in the UK [13] reporting a sound public awareness of oral cancer. The present study found a striking lack of knowledge about signs and symptoms of oral cancer among the study participants, despite the high incidence of oral cancer and its mortality rate in Teesside. The low awareness of early signs of oral cancer in high risk people has been also reported by others [13].

Lack of awareness of the disease has been suggested as a primary contributing factor in failure to seek help [9]. The present study showed that, as awareness of the existence of oral cancer was so low, respondents would not think to check their mouth for any signs or symptoms of it, nor indeed would they know what to look for. Thus a developing condition within the mouth would have to be very invasive or painful to be noticed. The benefits of oral cancer screening or regular mouth check-ups in reducing mortality and morbidity could be a powerful motivating factor in increasing screening uptake. The majority of participants in this study claimed that they would seek help, either immediately or within a few weeks, if they became aware of the signs. However, it was their GP rather than their dentist from whom they would seek help. They were not aware that dentists could undertake an oral cancer screening at routine check-up, and therefore they did not appreciate the role of dentists in screening for oral cancer. This was the case even for the few who regularly attended dental appointments. This was mainly due to the perception that: i) a dentist is ‘inaccessible’ on a physical (‘not registered’) and psychological (‘not welcome’; ‘fear’) level, ii) a check-up at the dentist will ‘cost’, unlike a doctor’s check-up, which is ‘free’, iii) a dentist is a ‘tooth specialist’, not a ‘mouth specialist’, and thus does not have the expertise to check for signs and symptoms of oral cancer, unlike a GP who has widespread expertise in examining for signs of cancer in all areas of the body, and iv) a dentist lacks the power of the doctor to prescribe medication and make referrals to specialists. The distributed free mouth check-up voucher, in this study, did not encourage the participants to attend the screening at the dental practice. The mind-set of participants towards mistrusting dentists, although not unanimous in the sample, would be a major challenge to convince them to be screened for oral cancer in a dental surgery.

An Oral Cancer Case-finding Intervention Project (OCCIP) was recently piloted elsewhere in the North East region (Gateshead and Newcastle) to promote early detection of oral cancer. The OCCIP was developed using the information obtained during a series of focus group studies with the target population to identify levels of oral cancer awareness and possible barriers to engagement with health care services. In this intervention, 205 vouchers were given out to ‘risk assessed’ recipients for free mouth checks at participating dental practices, of which 50 were subsequently redeemed when participants made appointments [14]. The low uptake rate of 24% in this intervention, despite the opportunity for a free dental check-up, further supports the findings of this study that the dental practice setting may not be the most suitable setting for an oral cancer screening programme.

The West of Scotland Cancer Awareness Project (WoSCAP) was also aimed at increasing public awareness and knowledge of mouth cancer and aimed to encourage early detection of symptoms among an at-risk population utilising a mass media approach [15]. Although the campaign was successful in utilising TV and publicity to increase awareness of the disease, and its symptoms, the impact was a short-term rather than a long-term increase in people accessing oral health checks. It was also found that patients attending rapid access clinics had often initially consulted their GP, as opposed to their dentist.

The participants in the present study believed a face-to-face conversation about oral cancer with a medical professional would have far greater impact than a media campaign. A powerful motivating factor for increasing oral screening uptake, therefore, could be employing a screening model and health professionals whom patients are already familiar with. Attendance at general medical practice for lifestyle advice and health-related screening such as cardiovascular screening is well accepted by patients. This “health intervention” opportunity could be used to promote a discussion about common risk factors i.e. smoking and drinking in relation to oral cancer. The offer of screening could be sent to at-risk individuals utilising the existing routes such as a letter from their GP. Therefore, medical appointments could be an efficient means of generating awareness of oral cancer and also offer the opportunity to be screened. The choice of screening location would appear to be a key factor in improving screening uptake. Screening locations that could be considered include GP surgeries, health centres, NHS workplace health assessments, and NHS branded mobile screening services. This would align the “offer” of oral cancer screening with other well accepted health screening services and address the concerns of participants about screening being a “business” opportunity for the dentist, as opposed to a health benefit for patients.

The participants in this research displayed a reticence to be told what or what not to do, and they revealed a propensity to feel ‘victimised’ and ‘singled out’ by health campaigns for being smokers/drinkers. Therefore an important element of encouraging them to take part in oral cancer screening could be to adopt a non-judgemental and open policy, whereby all men over 40 years of age, no matter what their lifestyle habits, are invited to attend.

Additionally, there may be value in emphasising the potential for heavy smoker/drinkers to have more agency themselves, rather than seeing them as entirely dependent on professionals for safeguarding their health. A recent review [16] showed that up to three-quarters of ex-smokers managed to quit unaided and suggested that health authorities should emphasise the positive message that the most successful methods used by ex-smokers were “cold turkey” and “reducing-then-quitting”. An approach which delivered motivational messages (perhaps through informative leaflets giving successful stories and outcome statistics) designed to encourage them to discontinue the main risk factors (drinking and smoking), which promoted/stimulated screening, and which gave easy access for direct conversation with a medical professional could be used to improve both quit rates and screening for oral cancers.

In conclusion, the uptake of oral cancer screening may be improved by increasing knowledge of the existence and signs and symptoms of oral cancer. Other factors that may increase uptake are: increased awareness of the role of dentists in diagnosing oral cancer, promotion of oral cancer screening by health professionals during routine health checks, and the choice of a “health” screening setting as opposed to a “dental” setting.
